# Neonatal Respiratory Care

**DOI:** 10.1155/2012/213974

**Published:** 2012-05-15

**Authors:** Mei-Jy Jeng, Tsu F. Yeh, Peter A. Dargaville, Thomas H. Shaffer, Jen-Tien Wung

**Affiliations:** ^1^Institute of Emergency and Critical Care Medicine and Department of Pediatrics, School of Medicine, National Yang-Ming University, Taipei 11221, Taiwan; ^2^Department of Pediatrics, Taipei Veterans General Hospital, Taipei 11217, Taiwan; ^3^Department of Pediatrics, University of Illinois, Chicago, IL 60637, USA; ^4^Department of Pediatrics, Taipei Medical University, Taipei 11031, Taiwan; ^5^China Medical University, Taichung 40402, Taiwan; ^6^Neonatal and Paediatric Intensive Care, Royal Hobart Hospital, Hobart TAS 7000, Australia; ^7^Menzies Research Institute Tasmania and University of Tasmania, Hobart TAS 7000, Australia; ^8^Department of Pediatrics, Thomas Jefferson University, Phildelphia, PA 19107, USA; ^9^Department of Emeritus of Physiology and Pediatrics, Temple University, Phildelphia, PA 19122, USA; ^10^The Center For Pediatric Research and Nemours Research Lung Center and Office of Technology Transfer, A. I. duPont Hospital for Children, Wilmington, DE 19803, USA; ^11^Department of Pediatrics, Morgan Stanley Children's Hospital of New York, Columbia University Medical Center, New York, NY 11203, USA

Acute respiratory failure caused by different origins continues to be the major etiology of morbidity and mortality in critical neonates. There has been much advancement in neonatal respiratory care, but a few neonates with severe respiratory failure continue to be candidates for extracorporeal membrane oxygenation or survive with chronic lung diseases (CLD). Therefore, searching for ideal respiratory care strategies to reduce the morbidity and mortality rates is crucial for caring critical neonates. The main aim of this special issue was focused on the existing and potential strategies or techniques in neonatal respiratory care. In this special issue, we have invited a few papers that address such issues.

Meconium aspiration syndrome (MAS) is a common cause of severe respiratory failure in term infants. The associations of persistent pulmonary hypertension of newborn (PPHN), pulmonary air leaks, and other morbidities sometimes make the respiratory care a difficult challenge to neonatologists. Three papers address current respiratory care in MAS. In a paper by K. Swarnam et al. they have a detail review on the epidemiology, pathophysiology, and managements in many different views of MAS. In another paper, P. Dargaville focuses on the application of mechanical respiratory supports in MAS, as well as the role of adjunctive respiratory therapies. In a paper, by C. Fischer et al., they demonstrate the epidemiology of MAS in term neonates using a population-based retrospective study for all births from 2000 to 2007 in a French region (Burgundy).

 In addition, bronchopulmonary dysplasia (BPD)/chronic lung disease (CLD) continues to be a major cause of neonatal morbidity in spite of significant progress in the treatment of preterm neonates. We have two articles discussing the pharmacologic approaches for prevention and treatment of BPD/CLD. S. Gupta et al. focus on the use of corticosteroids in one paper, and K. Tropea reviews all possible medications and the future potential stem cell therapy in another one.

Furthermore, research papers discussing other important clinical issues in neonatal respiratory care are included. In a paper by S. Rastogi et al., they report their analysis on the factors associated with the successful weaning from nasal continuous positive airway pressure (NCPAP). In a paper by A. Gentili et al., they report their analysis on the duration of preoperative stabilization in predicting outcome of congenital diaphragmatic hernia. In a paper by K. Hole et al., they report the impact of neonatal resuscitation training in neonatal mortality rates in Malawi, Africa.

**Mei-Jy Jeng** **Mei-Jy Jeng** **Tsu F. Yeh** **Tsu F. Yeh** **Peter A. Dargaville** **Peter A. Dargaville** **Thomas H. Shaffer** **Thomas H. Shaffer** **Jen-Tien Wung** **Jen-Tien Wung**

